# Cook Co. Medical Society

**Published:** 1852-07

**Authors:** 


*Cook County Medical Society.*

JULY 6th.—Dr. Herrick remarked that there existed among authors a discrepancy in regard to the nature and treatment of Erysipelas—the subject for the evening's discussion,—that in his remarks he should speak from personal observation. Has noticed two forms of the disease, sthenic and asthenic,—has seen the first in New England,—believes that it is not dependent on internal causes, or at least on a specific poison. The disease as it exists in the East is the disease of authors,—has seen much benefit in those cases from external applications, protecting the surface from air,—thinks the cause of this variety doubtful, the *methodus medendi* obscure. In the West has more generally seen the asthenic form,—believes miasmatic influences give character to this affection,—thinks the chill and fever, coated tongue, high colored urine, and deficient action of the skin, indicate a torpidity of the excreting organs. Effete matter accumulates in the system with a tendency to purely chemical instead of chemico-vital changes.

The disease involves tissues possessed of low vitality, in which the life force is weak,—the heat following the chill may be the result of decomposing changes. The good effect of tonics and stimulants, combined with opiates, for the purpose of sustaining vital action and quieting irritability, is interesting in connection with this view of the subject,—thinks quinine, the tonic generally used, may act chemically, or by exciting the excreting organs,—brandy and other stimulants often useful,—thinks opium allays irritability by diminishing the circulation and respiration,—has obtained benefit from the use of the remedy in proportion as these effects were accomplished. Has two cases now in mind showing the value of this course of treatment:

CASE 1.—Mr. P., of Racine, was taken with a chill, followed by fever, with swelling of the parotid glands, which soon spread over the face and head—pulse rapid, skin hot—delirious,—had been treated with alteratives and antiphlogistics, at the time I saw him the pulse was 140 per minute, the extremities cool, nails blue, stertor and delirium. He was ordered brandy freely, with quinine, grs. v. every three or four hours.—He soon rallied and finally recovered.

Case 2.—Mr. M. P., admitted to the U. S. M. Hospital for a compound fracture of the lower portions of the fibula and tibia. Soon after he came in swelling extended upwards to the knee, the surface was red and painful,—he was ordered porter, with quinine grs. viij, opii grs. iij, every five hours till he should be completely under the influence of the medicine,—the pain almost immediately subsided, the spread of the inflammation was arrested. In this case the disorganising force was so great that an extensive slough of the cellular tissue formed, leaving a sinus which has not yet filled up. In this instance the disease may have been, and probably was, dependent on a specific poison, nevertheless it yielded promptly to stimulants and tonics.

Dr. Morfit remarked that the views expressed corresponded with the doctrines of Dr. Hamilton Bell, who believes that in this disease the capillaries lose their tonicity and become distended. Dr. Bell gives mur. tinc. iron, for the purpose of restoring tone to the vessels, and thus relieving them of their engorgement. May not stimulants and tonics act in the same way?

Dr. Davis—Has recognized two varieties—thinks they often run into each other,—are dependent on two sets of causes acting separately or conjointly,—one set of causes internal, probably retained excretable matters, another external, and similar to that giving rise to bilious fever,—has seen, in New York, the disease with symptoms very similar to those of fever,—this variety yielded very generally to an alterative and moderately antiphlogistic treatment. In the West has seen the disease in a different form, believes that here both external and internal causes are frequently combined in their action,—thinks that the disease as it appears in hospitals dependent on animal poisons, in such cases the system does not bear depletion,—believes all varieties of the disease may occur at the same time and place, as shown by the following cases:

Case 1.—A Norwegian of strong constitution and previous good health, had during the last winter an attack of erysipelas, characterized by high arterial excitement, a full strong pulse; moderately antiphlogistic treatment was used, and the disease soon yielded.

Case 2.—A. H., a patient in the Illinois Gen. Hospital, had been under treatment for some months for diabetes,—was very much emaciated, and the vital forces feeble. About the same time with the Norwegian she was attacked with an erysipelatous inflammation of one of the inferior extremities, the limb was œdematous to the knee, the surface red, hot and painful to the touch,—the entire surface was covered with collodion, and the patient ordered to take quin. grs. vj, opii grs. iij, every five or six hours, with carb. amm and camphor as stimulants,—the pain and swelling soon subsided, although a slough formed in the cellular tissue above the ancle which healed slowly.

Case 3.—A strong healthy girl, 10 years old, had previously suffered from inflammation, probably erysipelatous, of the pudendum. Previous to my seeing her she had been complaining of sore throat. I could only discover an unusual redness of the tonsils and fauces, in about two days she had a return of the disease over the pubes decidedly erysipelatous in its character,—while the face was perfectly healthy. Commenced immediately with antiphlogistics and alteratives, she soon recovered. An older sister was afterwards similarly attacked, and subjected to the same course of treatment, with a like termination.

In this disease quinine and opium are valuable remedies,—believes that calomel may generally be combined with them to advantage,—thinks there may be cases in which brandy is necessary, has never found such. Treatment in every instance should be adapted to the condition of the system rather than aimed at a name.

Dr. Mills remarked that in the early part of his professional life he saw only that variety characterized by high arterial excitement, and in which venesection, ant. and mercurials were borne, tonics only resorted to after convalescence was established,—has since used with most success blistering the surfaces and keeping the bowels open with some mild cathartic.

Dr. Palmer has seen much of the disease in an epidemic form, especially in puerperal women,—thinks it contagious and dependent on a specific poison,—has noticed in such cases a great tendency to depression. In the epidemic of 1844–5 the disease usually commenced with redness of the fauces, soreness of the throat, which rapidly spread to the integuments and adjacent mucous membranes. Diaphoretics, such as Dov. powders, and counter irritants, were most effectual in checking the disease—if these failed, vomiting soon commenced, inflammation extended to the bowels, and the patients mostly died,—the disease sometimes attacked the lungs, and in those cases almost always proved fatal. Phlegmonous erysipelas generally yielded to depletion in the first instance, followed by tonics after the inflammation was subdued,—puerperal cases mostly died, large bleedings and full doses of opium at the commencement of the attack were effectual in only a few instances, the bleeding had to be used early or not at all,—membranes of the brain and spinal cord sometimes involved, results fatal,—has generally found sporadic erysipelas amenable to antiphlogistic treatment,—thinks blisters most effectual as external applications, next tinc. iodine,—has used the collodion with success.

Dr. McArthur remarked that in high latitudes not malarious, the idea prevailed that the disease is not contagious, in low malarious districts the contrary opinion is entertained,—has seen both varieties of the disease, the sthenic and asthenic, the former in the state of N. York, and the latter in the west.

On motion, the further discussion of the subject was deferred till the next regular meeting of the society, and Dr. Davis requested to report on the specific nature and communicability of erysipelas, especially as it occurs in puerperal women.

Dr. McArthur reported the following case, which gave rise to an interesting discussion :—

May 4. I was called to see Mrs. J. B. B., of Lasalle Co., of this State, and found her at her sister's residence, Mrs. Perkin's on Madison-street. She informed me that she was taken suddenly ill, in March, 1851, with general symptoms of cold, such as cough, rheumatic pains of the limbs and joints, general prostration, &c., together with inflammation of the eye-lids and the balls of the eyes, or in other words general ophthalmia. She was treated by several practitioners in turn (five or more) from that time until a short time previously to her coming to this city.

She stated that she had been confined to her bed and rocking chair most of the time since her attack, and had been unable to distinguish (by seeing) one of her family from another since last October.

For several months, for the most or all of the time, she had been unable to walk without assistance, and had not had a good night's rest since last fall. Her appetite was very poor, and her menstrual evacuations excessive and accompanied with leucorrhœa.

She informed me, furthermore, that she had had one seton inserted in the back of the neck, and twenty-eight blister plasters applied in different localities, most of them being quite large, that she had local applications of different kinds for the eyes, and had submitted to general treatment. For some months previous to her coming here, she had almost constantly in the day time (when the eye was bare, and the light impinged upon it) discovered a semblance or appearance of balls of fire constantly passing before the left eye. The same had been discovered with the right eye, only to a less extent.

One of her physicians informed her a few days before she left home (not knowing of her intention to come to Chicago,) that she would be entirely blind in some four weeks, and would never again see—that her case was hopeless.

In coming here, she was two days in passing from her residence to the canal at Ottawa, a distance not exceeding twelve miles. And finally she was almost in despair, fearing she should never be able to see again.

I found her pulse feeble, her muscular system flabby, tongue covered with light brown fur, the back part of the mouth inflamed, uvula greatly inflamed and elongated, and the patient was quite deaf.

The conjunctivæ were inflamed over the balls of the eye, and over the whole of their attachment to the upper and lower lids, and in their connection to the carunculæ lacrymales. A film extended over the left eye, almost obscuring the cornea. The blood vessels, extending from the inner canthus of each eye, could be distinctly seen passing over the entire cornea.

The muscles of the eyelids seemed to be *almost* paralyzed—there being very *little* power over the movements of the lids; and the muscles of the ball seemed to be *completely* paralyzed—there being *no* power, by an effort of the will, to move the balls one way or the other.

The eyelids were adhered together, and required force to bring them apart, or even to keep them so after they were parted.

In the progress of my examination of the case, there were indications of rheumatic ophthalmia and iritis, and in the progress of the treatment this became still more apparent than at first.

TREATMENT.—I shall be brief in regard to the treatment of the case, for the most part giving the general outline only.

I first amputated the uvula, and then directed the application of nitrate of silver in solution (of the strength of grs. vj to the ℥j of water,) to the throat, by means of the probang.

Pills of hydrarg. were given a few times, to be followed with castor oil or Turkey rhubarb, sufficient to produce moderate catharsis.

Having become fully satisfied that a rheumatic diathesis prevailed, I endeavored to administer such agents as were calculated to remove or modify this condition of the system.

The local treatment to the eyes has been varied from time to time, as the exigencies of the case seemed to require. The blood vessels passing over the balls were most of them incised. The lids have been scarified with a thumb lance; and nitrate of silver, the sulphates of copper and zinc, have been applied in substance and in solution, of different degrees of strength, to the inflamed conjunctivæ.

The solutions have been applied sometimes with the camel's hair pencil, and at other times dropped into the eyes, the lids being held open.

Mercurial ointment has been applied on the outside of the lids at various times.

The patient was directed to drink ale twice or more times daily, for five weeks continuously, and occasionally a little port wine.

Beef steak and mutton chop were not prohibited even from the first day I prescribed for her. And when her general strength had improved sufficiently, and the weather would permit, she was directed to walk a short time in the open air, morning and evening, about the yard,—some person leading her.

PRESENT CONDITION.—Her health and strength are sufficient for her to walk from half a mile to a mile, morning and evening, without being weary or suffering inconvenience therefrom. Her appetite is now generally good, and her sleep is quiet and invigorating.

The inflammation of the conjunctivæ in their connection with the balls of the eyes, is entirely removed, and principally so in their connection with the lids, and the carunculæ lachrymales.

The film which was so well developed over the cornea of the left eye, is apparently removed; and the blood vessels which were so extended and enlarged over both eyes, have become reduced almost to their normal condition. The muscles of the balls have resumed their original actions of contractions and relaxations, so that the balls of the eyes move again with facility, and the lids are moved with ease and freedom.

She can now read twenty or thirty minutes continuously without any other inconvenience than perhaps a slight pain of the muscles of the eyes, or, as she expresses it, "the eyes feel tired." She has sewed some at different times lately, for the first since her attack; and for the last two or three weeks has corresponded with her husband.

She has entirely recovered from her deafness.

Hope of life and its enjoyments has returned to a heart almost given over to despair, and the fond expectation is indulged of again *seeing* her husband and children, and enjoying their society.

---

*Knox County Medical Society.*

